# Supplementary material for: Inhibitory Effect and Mechanism of Action of Quercetin and Quercetin Diels-Alder anti-Dimer on Erastin-Induced Ferroptosis in Bone Marrow-Derived Mesenchymal Stem Cells
Source: Antioxidants (Basel). 2020 Mar 2;9(3):205. doi: 10.3390/antiox9030205 (PMC7139729; doi:10.3390/antiox9030205)
Supplement: Supplementary file 1 [file antioxidants-09-00205-s001.zip › antioxidants-715022-supplementary files-final/Suppl 1 latest studies of quercetin on various cells (2018-2019).pdf]

Suppl. 1 The list of latest studies of quercetin on various cells (2018-2019).

[1] Maurya, A.K.; Vinayak, M. Improved synergistic anticancer efficacy of quercetin in combination with PI-103, rottlerin, and G0 6983 against MCF-7 and RAW 264.7 cells. *In Vitro Cell Dev Biol Anim*. **2019**, 55, 36-44.

[2] Jazvinscak Jembrek, M.; Vlainic, J.; Cadez, V.; Segota, S. Atomic force microscopy reveals new biophysical markers for monitoring subcellular changes in oxidative injury: Neuroprotective effects of quercetin at the nanoscale. *PLoS One*. **2018**, 13, e0200119.

[3] Houghton, M.J.; Kerimi, A.; Tumova, S.; Boyle, J.P.; Williamson, G. Quercetin preserves redox status and stimulates mitochondrial function in metabolically-stressed HepG2 cells. *Free Radic Biol Med*. **2018**, 129, 296-309.

[4] Lee, Y.J.; Beak, S.Y.; Choi, I.; Sung, J.S. Quercetin and its metabolites protect hepatocytes against ethanol-induced oxidative stress by activation of Nrf2 and AP-1. *Food Sci Biotechnol*. **2018**, 27, 809-817.

[5] Rubio, V.; Garcia-Perez, A.I.; Herraez, A.; Diez, J.C. Different roles of Nrf2 and NFKB in the antioxidant imbalance produced by esculetin or quercetin on NB4 leukemia cells. *Chem Biol Interact*. **2018**, 294, 158-166.

[6] Postaci, I.; Coskun, O.; Senol, N.; Aslankoc, R.; Comlekci, S. The physiopathological effects of quercetin on oxidative stress in radiation of 4.5 g mobile phone exposed liver tissue of rat. *Bratisl Lek Listy*. **2018**, 119, 481-489.

[7] Chen, Z.; Yuan, Q.; Xu, G.; Chen, H.; Lei, H.; Su, J. Effects of Quercetin on Proliferation and H(2)O(2)-Induced Apoptosis of Intestinal Porcine Enterocyte Cells. *Molecules*. **2018**, 23,

[8] Chirumbolo, S.; Bjorklund, G. The purported kinship between melatonin and quercetin in the anti-oxidant activity against the LPS-mediated cell damage. *Food Chem Toxicol*. **2018**, 120, 588-589.

[9] Rastogi, S.; Haldar, C. Comparative effect of melatonin and quercetin in counteracting LPS induced oxidative stress in bone marrow mononuclear cells and spleen of Funambulus pennanti. *Food Chem Toxicol*. **2018**, 120, 243-252.

[10] Wang, J.; Qian, X.; Gao, Q.; Lv, C.; Xu, J.; Jin, H.; Zhu, H. Quercetin increases the antioxidant capacity of the ovary in menopausal rats and in ovarian granulosa cell culture in vitro. *J Ovarian Res*. **2018**, 11, 51.

[11] Jin, Y.; Huang, Z.L.; Li, L.; Yang, Y.; Wang, C.H.; Wang, Z.T.; Ji, L.L. Quercetin attenuates toosendanin-induced hepatotoxicity through inducing the Nrf2/GCL/GSH antioxidant signaling pathway. *Acta Pharmacol Sin*. **2019**, 40, 75-85.

[12] Ward, A.B.; Mir, H.; Kapur, N.; Gales, D.N.; Carriere, P.P.; Singh, S. Quercetin inhibits prostate cancer by attenuating cell survival and inhibiting anti-apoptotic pathways. *World J Surg Oncol*. **2018**, 16, 108.

[13] Abdel Aziz, R.L.; Abdel-Wahab, A.; Abo El-Ela, F.I.; Hassan, N.E.Y.; El-Nahass, E.S.; Ibrahim, M.A.; Khalil, A.A.Y. Dose- dependent ameliorative effects of quercetin and l-Carnitine against atrazine- induced reproductive toxicity in adult male Albino rats. *Biomed Pharmacother*. **2018**, 102, 855-864.

[14] Alugoju, P.; Periyasamy, L.; Dyavaiah, M. Quercetin enhances stress resistance in Saccharomyces cerevisiae tel1 mutant cells to different stressors. *J Food Sci Technol*. **2018**,

55, 1455-1466.
[15] Boadi, W.Y.; Lo, A. Effects of Quercetin, Kaempferol, and Exogenous Glutathione on Phospho- and Total-AKT in 3T3-L1 Preadipocytes. *J Diet Suppl*. **2018**, 15, 814-826.
[16] Chan, S.T.; Chuang, C.H.; Lin, Y.C.; Liao, J.W.; Lii, C.K.; Yeh, S.L. Quercetin enhances the antitumor effect of trichostatin A and suppresses muscle wasting in tumor-bearing mice. *Food Funct*. **2018**, 9, 871-879.
[17] Guo, X.; Chen, M.; Zeng, H.; Liu, P.; Zhu, X.; Zhou, F.; Liu, J.; Zhang, J.; Dong, Z.; Tang, Y.; Gao, C.; Yao, P. Quercetin Attenuates Ethanol-Induced Iron Uptake and Myocardial Injury by Regulating the Angiotensin II-L-Type Calcium Channel. *Mol Nutr Food Res*. **2018**, 62,
[18] Bonechi, C.; Donati, A.; Tamasi, G.; Leone, G.; Consumi, M.; Rossi, C.; Lamponi, S.; Magnani, A. Protective effect of quercetin and rutin encapsulated liposomes on induced oxidative stress. *Biophys Chem*. **2018**, 233, 55-63.
[19] Eftekhari, A.; Ahmadian, E.; Panahi-Azar, V.; Hosseini, H.; Tabibiazar, M.; Maleki Dizaj, S. Hepatoprotective and free radical scavenging actions of quercetin nanoparticles on aflatoxin B1-induced liver damage: in vitro/in vivo studies. *Artif Cells Nanomed Biotechnol*. **2018**, 46, 411-420.
[20] Boumaza, S.; Belkebir, A.; Neggazi, S.; Sahraoui, H.; Berdja, S.; Smail, L.; Benazzoug, Y.; Kacimi, G.; Aouichat Bouguerra, S. Therapeutic Role of Resveratrol and Quercetin on Aortic Fibroblasts of Psammomys obesus After Oxidative Stress by Hydrogen Peroxide. *Am J Ther*. **2018**, 25, e299-e313.
[21] Sun, G.Y.; Li, R.; Yang, B.; Fritsche, K.L.; Beversdorf, D.Q.; Lubahn, D.B.; Geng, X.; Lee, J.C.; Greenlief, C.M. Quercetin Potentiates Docosahexaenoic Acid to Suppress Lipopolysaccharide-induced Oxidative/Inflammatory Responses, Alter Lipid Peroxidation Products, and Enhance the Adaptive Stress Pathways in BV-2 Microglial Cells. *Int J Mol Sci*. **2019**, 20,
[22] Rezabakhsh, A.; Rahbarghazi, R.; Malekinejad, H.; Fathi, F.; Montaseri, A.; Garjani, A. Quercetin alleviates high glucose-induced damage on human umbilical vein endothelial cells by promoting autophagy. *Phytomedicine*. **2019**, 56, 183-193.
[23] Alidadi, H.; Khorsandi, L.; Shirani, M. Effects of Quercetin on Tubular Cell Apoptosis and Kidney Damage in Rats Induced by Titanium Dioxide Nanoparticles. *Malays J Med Sci*. **2018**, 25, 72-81.
[24] Zhu, Q.; Liu, M.; He, Y.; Yang, B. Quercetin protect cigarette smoke extracts induced inflammation and apoptosis in RPE cells. *Artif Cells Nanomed Biotechnol*. **2019**, 47, 2010-2015.
[25] Niazvand, F.; Orazizadeh, M.; Khorsandi, L.; Abbaspour, M.; Mansouri, E.; Khodadadi, A. Effects of Quercetin-Loaded Nanoparticles on MCF-7 Human Breast Cancer Cells. *Medicina (Kaunas)*. **2019**, 55,
[26] Cebecioglu, R.; Yildirim, M.; Akagunduz, D.; Korkmaz, I.; Tekin, H.O.; Atasever-Arslan, B.; Catal, T. Synergistic effects of quercetin and selenium on oxidative stress in endometrial adenocarcinoma cells. *Bratisl Lek Listy*. **2019**, 120, 449-455.
[27] Zhang, P.; Mak, J.C.; Man, R.Y.; Leung, S.W. Flavonoids reduces lipopolysaccharide-induced release of inflammatory mediators in human bronchial epithelial cells: Structure-activity relationship. *Eur J Pharmacol*. **2019**, 865, 172731.

[28] Tian, R.; Yang, Z.; Lu, N.; Peng, Y.Y. Quercetin, but not rutin, attenuated hydrogen peroxide-induced cell damage via heme oxygenase-1 induction in endothelial cells. *Arch Biochem Biophys*. **2019**, 676, 108157.

[29] Tang, J.; Diao, P.; Shu, X.; Li, L.; Xiong, L. Quercetin and Quercitrin Attenuates the Inflammatory Response and Oxidative Stress in LPS-Induced RAW264.7 Cells: In Vitro Assessment and a Theoretical Model. *Biomed Res Int*. **2019**, 2019, 7039802.

[30] Salama, Y.A.; El-Karef, A.; El Gayyar, A.M.; Abdel-Rahman, N. Beyond its antioxidant properties: Quercetin targets multiple signalling pathways in hepatocellular carcinoma in rats. *Life Sci*. **2019**, 236, 116933.

[31] Rashidi, Z.; Aleyasin, A.; Eslami, M.; Nekoonam, S.; Zendedel, A.; Bahramrezaie, M.; Amidi, F. Quercetin protects human granulosa cells against oxidative stress via thioredoxin system. *Reprod Biol*. **2019**, 19, 245-254.

[32] Lewinska, A.; Adamczyk-Grochala, J.; Bloniarz, D.; Olszowka, J.; Kulpa-Greszta, M.; Litwinienko, G.; Tomaszewska, A.; Wnuk, M.; Pazik, R. AMPK-mediated senolytic and senostatic activity of quercetin surface functionalized Fe_3O_4 nanoparticles during oxidant-induced senescence in human fibroblasts. *Redox Biol*. **2020**, 28, 101337.

[33] Khan, H.; Ullah, H.; Aschner, M.; Cheang, W.S.; Akkol, E.K. Neuroprotective Effects of Quercetin in Alzheimer's Disease. *Biomolecules*. **2019**, 10,

[34] Granato, M.; Gilardini Montani, M.S.; Zompetta, C.; Santarelli, R.; Gonnella, R.; Romeo, M.A.; D'Orazi, G.; Faggioni, A.; Cirone, M. Quercetin Interrupts the Positive Feedback Loop Between STAT3 and IL-6, Promotes Autophagy, and Reduces ROS, Preventing EBV-Driven B Cell Immortalization. *Biomolecules*. **2019**, 9,

[35] Brisdelli, F.; Di Francesco, L.; Giorgi, A.; Lizzi, A.R.; Luzi, C.; Mignogna, G.; Bozzi, A.; Schinina, M.E. Proteomic Analysis of Quercetin-Treated K562 Cells. *Int J Mol Sci*. **2019**, 21,

[36] Ahmed, O.M.; Ahmed, A.A.; Fahim, H.I.; Zaky, M.Y. Quercetin and naringenin abate diethylnitrosamine/acetylaminofluorene-induced hepatocarcinogenesis in Wistar rats: the roles of oxidative stress, inflammation and cell apoptosis. *Drug Chem Toxicol*. **2019**, 10.1080/01480545.2019.16831871-12.
