# Supplementary material for: Inhibitory Effect and Mechanism of Action of Quercetin and Quercetin Diels-Alder anti-Dimer on Erastin-Induced Ferroptosis in Bone Marrow-Derived Mesenchymal Stem Cells
Source: Antioxidants (Basel). 2020 Mar 2;9(3):205. doi: 10.3390/antiox9030205 (PMC7139729; doi:10.3390/antiox9030205)
Supplement: Supplementary file 1 [file antioxidants-09-00205-s001.zip › antioxidants-715022-supplementary files-final/Suppl 4 Photo and certificate analysis of QDAD.pdf]

Fig. S4.1 Photo of Quercetin Diels-Alder *anti*-Dimer

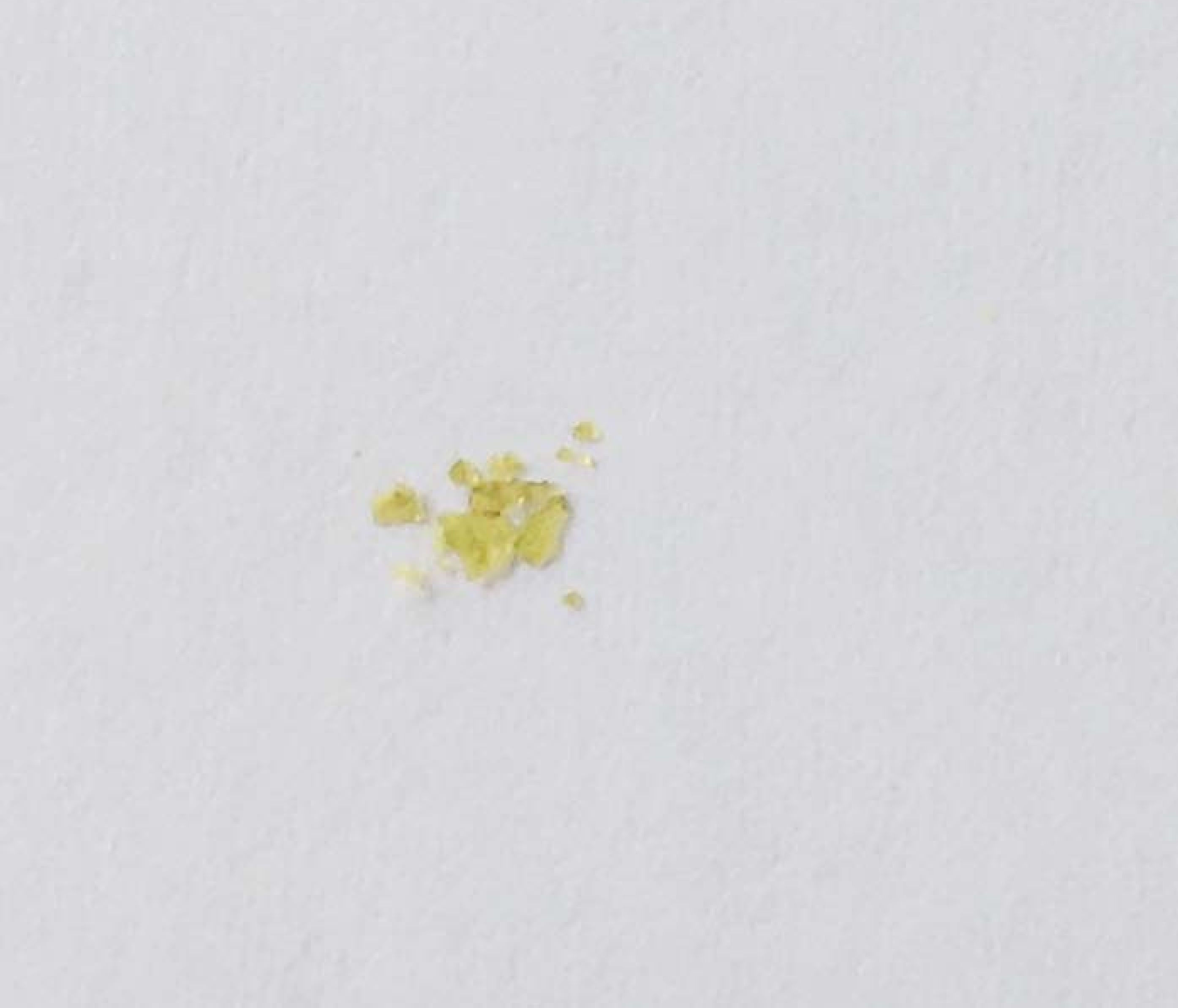

Fig. S4.2

西力生物
BBP BioBioPha
Natural Product Library for Drug Discovery

CERTIFICATE OF ANALYSIS

BBP No.: BBP05214

CAS No.: 167276-19-9

Chemical Name: Quercetin dimer

Molecular Formula: $C_{30}H_{18}O_{14}$

Structure:

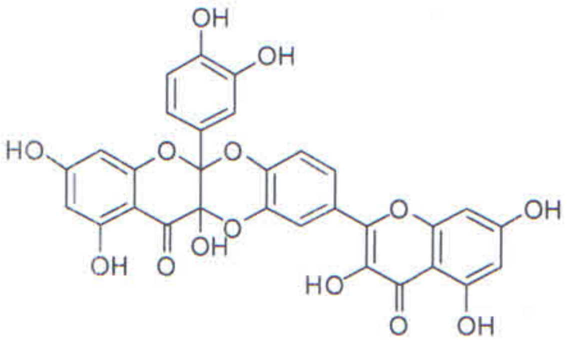

Purity: 98%

Appearance: Yellow cryst.

Solvent: Methanol

Exact Weight: 5.1 mg

Storage: Store in a dark place under the temperature of 0−4 °C

Intended Use: For laboratory use only

Reference: T. N. Ly, J Agric Food Chem, 2005, 53(21), 8183-8189

Warm Notice: When publishing, please cite as: ***chemical name*** was purchased from BioBioPha Co., Ltd. (Kunming, China)

Characterization Data Summary

Analytical Test	Results
Identification by ^{1}H-NMR	Consistent with the above structure
Purity tested by HPLC, ^{1}H-NMR	98%

Authorized Signature: BioBioPha Co., Ltd. 2019.02.26 PASS

Date:

Tel.: +86 871 6521 7109 Fax: +86 871 6521 5563 E-mail: sales@mail.biobiopha.com

Fig. S4.3 HPLC and UV

西力生物
BBP BioBioPha
Natural Product Library for Drug Discovery

PRODUCT QUALITY REPORT

Product Number: BBP05214

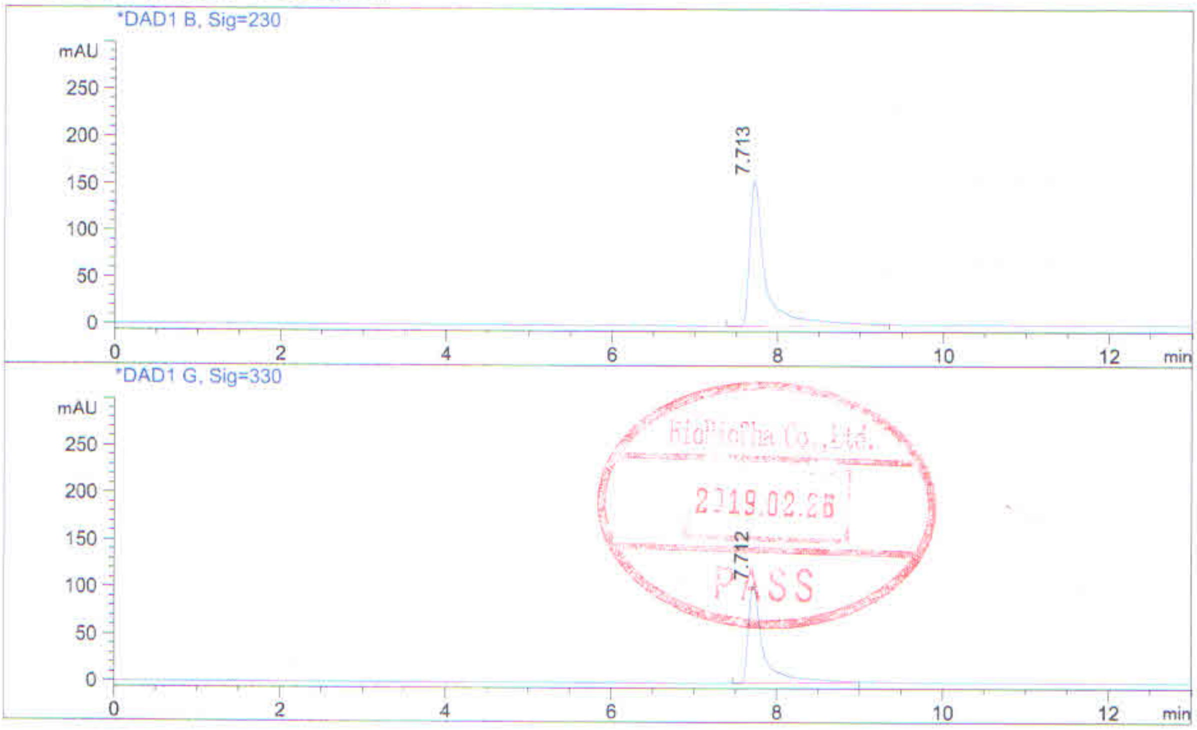

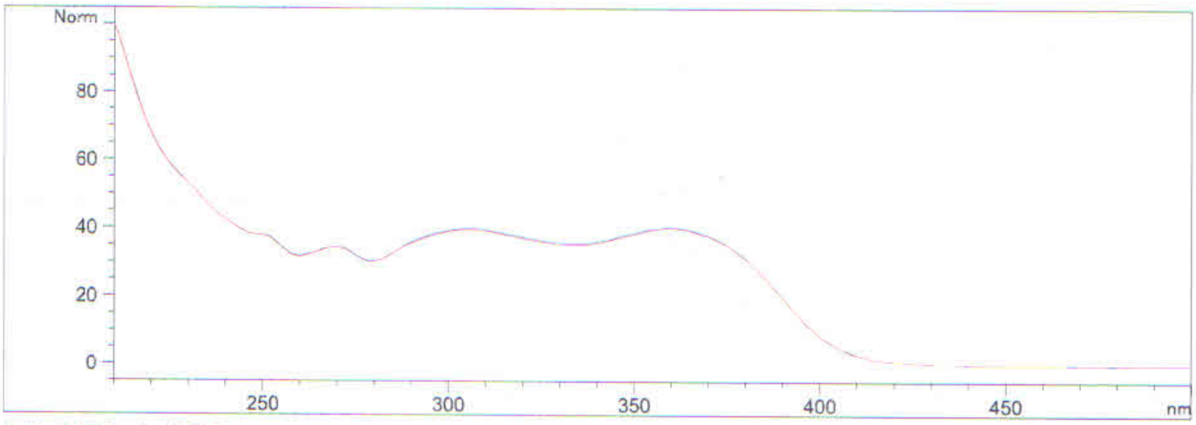

Agilent 1200 series HPLC system
Extend-C18 column (5 μm, 4.6 × 150 mm)
20%→100% MeOH in H_2O over 8.0 min followed by 100% MeOH to 13.0 min
1.0 ml/min, 20°C

Fig. S4.4 H - NMR

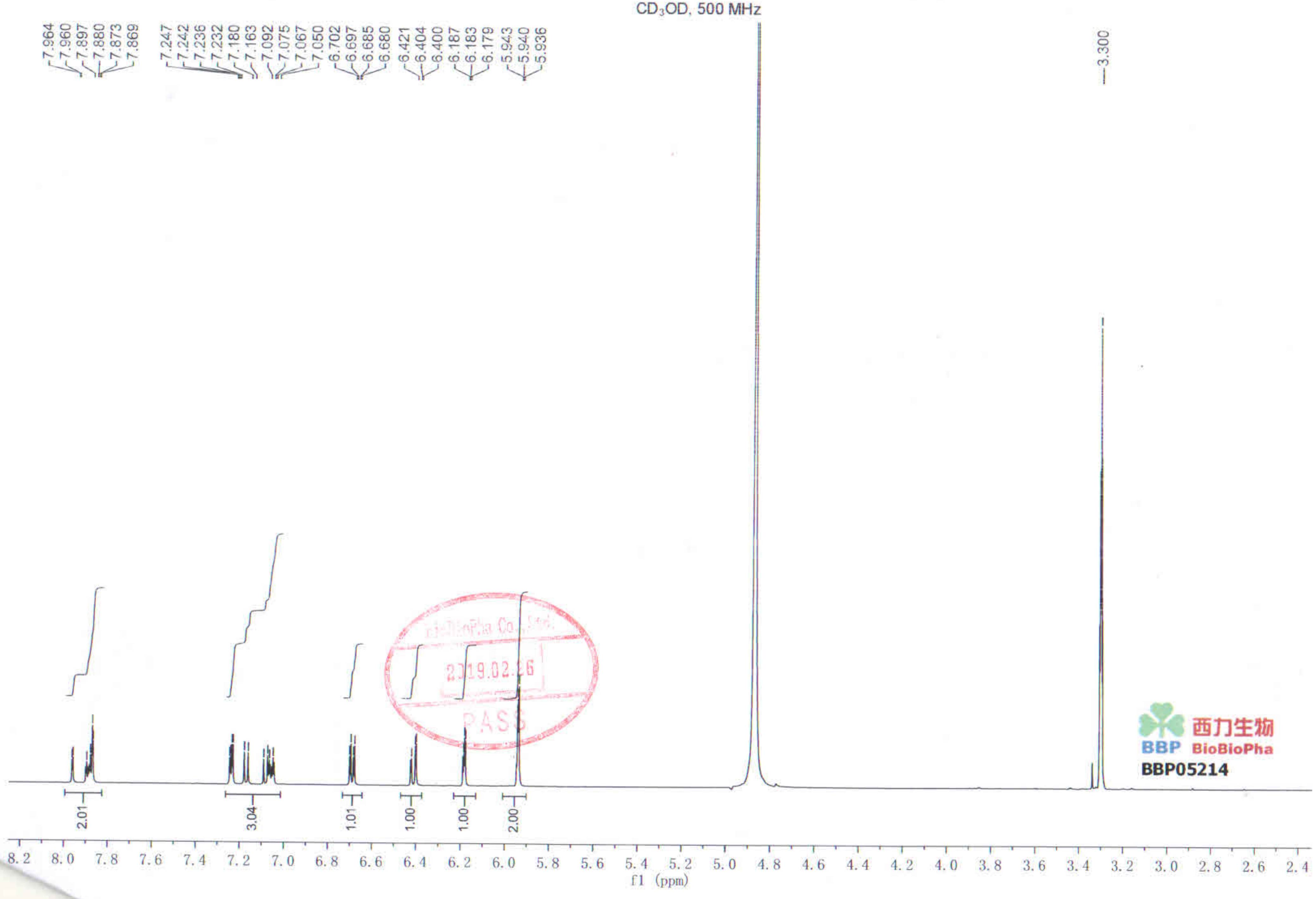
CD$_3$OD, 500 MHz
7.964
7.960
7.897
7.880
7.873
7.869
7.247
7.242
7.236
7.232
7.180
7.163
7.092
7.075
7.067
7.050
6.702
6.697
6.685
6.680
6.421
6.404
6.400
6.187
6.183
6.179
5.943
5.940
5.936
3.300
2.01
3.04
1.01
1.00
1.00
2.00
8.2 8.0 7.8 7.6 7.4 7.2 7.0 6.8 6.6 6.4 6.2 6.0 5.8 5.6 5.4 5.2 5.0 4.8 4.6 4.4 4.2 4.0 3.8 3.6 3.4 3.2 3.0 2.8 2.6 2.4
f1 (ppm)
2019.02.26
PASS
西力生物
BBP BioBioPha
BBP05214
