# Supplementary material for: Inhibitory Effect and Mechanism of Action of Quercetin and Quercetin Diels-Alder anti-Dimer on Erastin-Induced Ferroptosis in Bone Marrow-Derived Mesenchymal Stem Cells
Source: Antioxidants (Basel). 2020 Mar 2;9(3):205. doi: 10.3390/antiox9030205 (PMC7139729; doi:10.3390/antiox9030205)
Supplement: Supplementary file 1 [file antioxidants-09-00205-s001.zip › antioxidants-715022-supplementary files-final/Suppl 5 Dose¿Cresponse curves.pdf]

Suppl. 5 Concentration-response curves

Inhibitory Effect and Mechanism of Action of Quercetin and Quercetin Diels-Alder *anti*-Dimer on Erastin-induced Ferroptosis in Bone Marrow-derived Mesenchymal Stem Cells

Xican Li [1,2,*,†], Jingyuan Zeng [1,†], Yangping Liu[3], Minshi Liang [1,2], Qianru Liu[1,2], Zhen Li [4], Xiaojun Zhao [1,2], Dongfeng Chen[4,*]

[1] School of Chinese Herbal Medicine, Guangzhou University of Chinese Medicine, Waihuan East Road No. 232, Guangzhou Higher Education Mega Center, Guangzhou 510006, China; zengjingyuan710@163.com (J.Z.); lminshi@outlook.com (M.L.); zxj@gzucm.edu.cn (X.Z.)

[2] Innovative Research & Development Laboratory of TCM, Guangzhou University of Chinese Medicine, Waihuan East Road No. 232, Guangzhou Higher Education Mega Center, Guangzhou 510006, China;

[3] The Fourth Clinical Medical College, Guangzhou University of Chinese Medicine, Waihuan East Road No. 232, Guangzhou Higher Education Mega Center, Guangzhou 510006, China. dryangpingliu@163.com (Y.L.);

[4] Department of Anatomy, The Research center of Basic Integrative Medicine, Guangzhou University of Chinese Medicine, Guangzhou, Guangdong 510006, China; jantqleed@163.com (Z.L.); chen888@gzucm.edu.cn (D.C.);

* Correspondence: lixican@126.com (X.L.) or zml1930896811@163.com (M. Z.); Tel.: +86-203-935-8076 (X.L.)

† These authors contributed equally to this work.

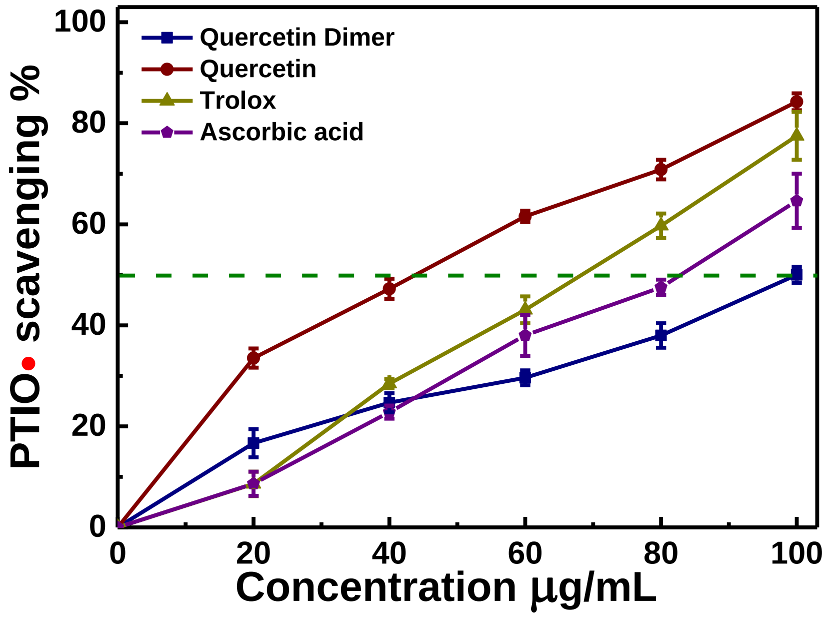

Figure s1.1. Concentration-response curves for quercetin, (±) quercetin Diels-Alder anti-dimer, Trolox and ascorbic acid in PTIO•-scavenging. Each value is expressed as the mean ± S, n = 3.

Table 1. The IC_{50} values of QDAD, quercetin, **Trolox** and **ascorbic acid** in PTIO•-scavenging antioxidant measurements

	QDAD	Quercetin	Trolox	Ascorbic acid
μg/mL	109.1±6.4	44.5±0.6	161.5±9.2	80.5±4.1
μM	181.2±10.9[b]	147.3±2.0[a]	646.0±98.2[d]	457.4±24.2[c]

The IC_{50} value is defined as the lowest concentration with 50% radical inhibition or relative reducing power, calculated by linear regression analysis, and expressed as the mean ± SD (*n* = 3). The linear regression was analyzed using Origin 6.0 professional software. The IC_{50} values in μM with different superscripts ([a] and [b]) in the same row are significantly different ($p < 0.05$). Trolox and ascorbic acid were used as the positive controls.

The data in Red are cited in Table 1 in the main text.

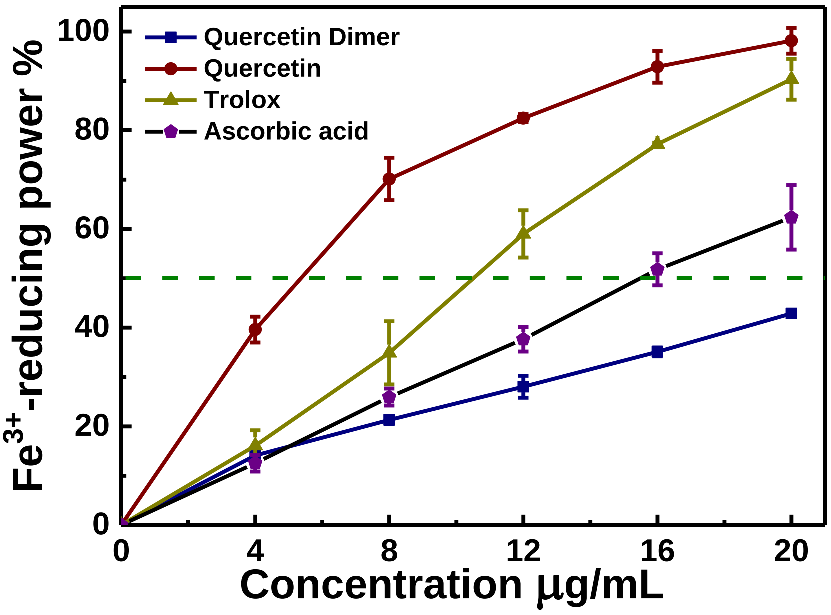

Figure s1.2. Concentration-response curves for quercetin, (±) quercetin Diels-Alder anti-dimer, Trolox and ascorbic acid in in FRAP antioxidant measurement. Each value is expressed as the mean ± S, n = 3.

Table 2. The IC_{50} values of QDAD, quercetin, **Trolox** and **ascorbic acid** in FRAP antioxidant measurement

	QDAD	Quercetin	Trolox	Ascorbic acid
µg/mL	24.1±0.6	4.6±0.3	10.8±0.7	16.1±1.4
µM	40.1±1.2 [b]	15.2±1.0 [a]	43.3±2.8[c]	91.1±8.1 [d]

The IC_{50} value is defined as the lowest concentration with 50% radical inhibition or relative reducing power, calculated by linear regression analysis, and expressed as the mean ± SD (n = 3). The linear regression was analyzed using Origin 6.0 professional software. The IC_{50} values in µM with different superscripts ([a] and [b]) in the same row are significantly different (p< 0.05). Trolox and ascorbic acid were used as the positive controls.
The data in Red are cited in Table 1 in the main text.

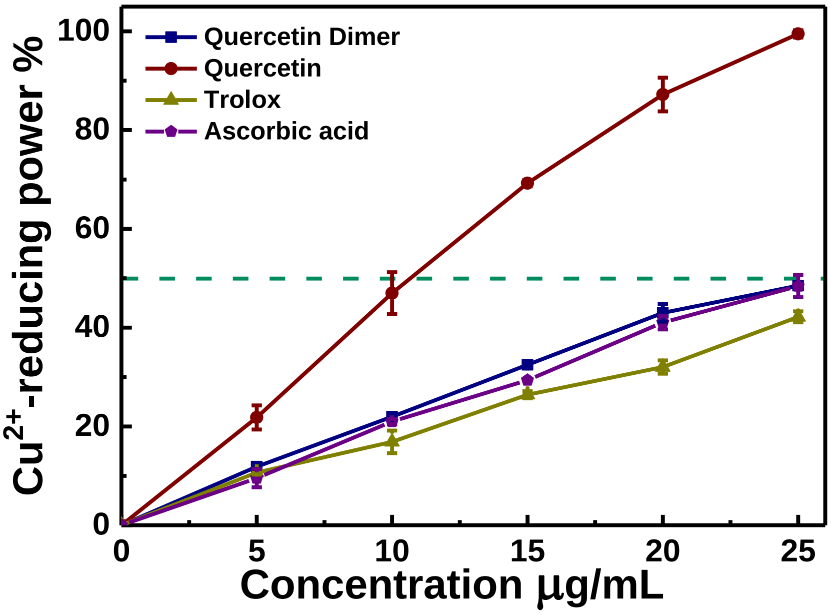

Figure s1.3. Concentration-response curves for quercetin, (±) quercetin Diels-Alder anti-dimer, Trolox and ascorbic acid in in CUPRAC antioxidant measurement. Each value is expressed as the mean ± S, n = 3.

Table 3. The IC_{50} values of QDAD, quercetin, **Trolox** and **ascorbic acid** in CUPRAC antioxidant measurement

	QDAD	Quercetin	Trolox	Ascorbic acid
μg/mL	26.1±0.6	11.3±0.9	28.8±2.9	32.3±3.2
μM	43.3±1.2 [b]	37.4±3.0 [a]	115.2±11.6[c]	183.5±8.2 [d]

The IC_{50} value is defined as the lowest concentration with 50% radical inhibition or relative reducing power, calculated by linear regression analysis, and expressed as the mean ± SD (n = 3). The linear regression was analyzed using Origin 6.0 professional software. The IC_{50} values in μM with different superscripts ([a] and [b]) in the same row are significantly different (p< 0.05). Trolox and ascorbic acid were used as the positive controls.

The data in Red are cited in Table 1 in the main text.

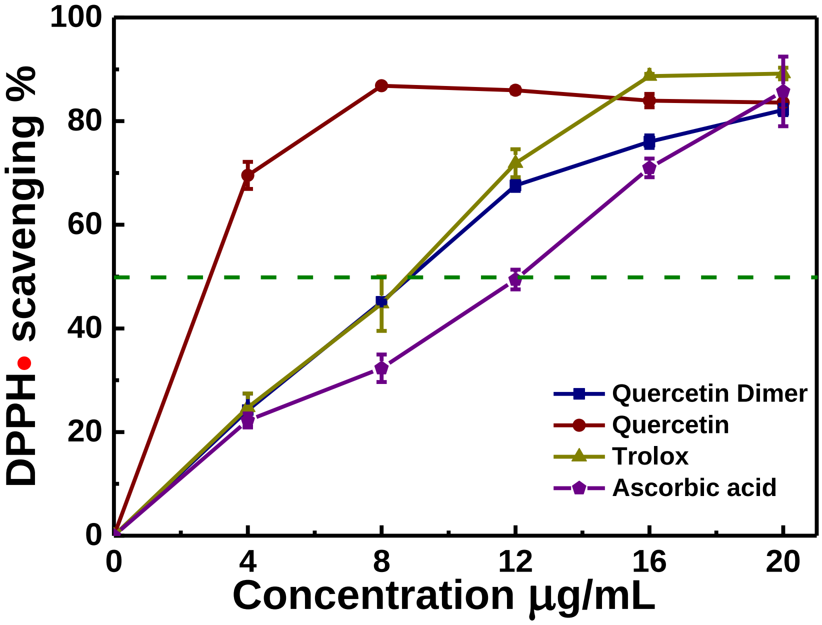

Figure s1.4. Concentration-response curves for quercetin, (±) quercetin Diels-Alder anti-dimer, Trolox and ascorbic acid in in CUPRAC antioxidant measurement. Each value is expressed as the mean ± S, n = 3.

Table 4. The IC_{50} values of QDAD, quercetin, Trolox and ascorbic acid in CUPRAC antioxidant measurement

	QDAD	Quercetin	Trolox	Ascorbic acid
µg/mL	12.2±1.2	3.3±0.3	8.9±1.2	9.1±0.3
µM	20.3±2.0[b]	10.9±1.1 [a]	35.6±4.5[c]	51.7±0.7 [d]

The IC_{50} value is defined as the lowest concentration with 50% radical inhibition or relative reducing power, calculated by linear regression analysis, and expressed as the mean ± SD (n = 3). The linear regression was analyzed using Origin 6.0 professional software. The IC_{50} values in µM with different superscripts ([a] and [b]) in the same row are significantly different ($p < 0.05$). Trolox and ascorbic acid were used as the positive controls.

The data in Red are cited in Table 1 in the main text.

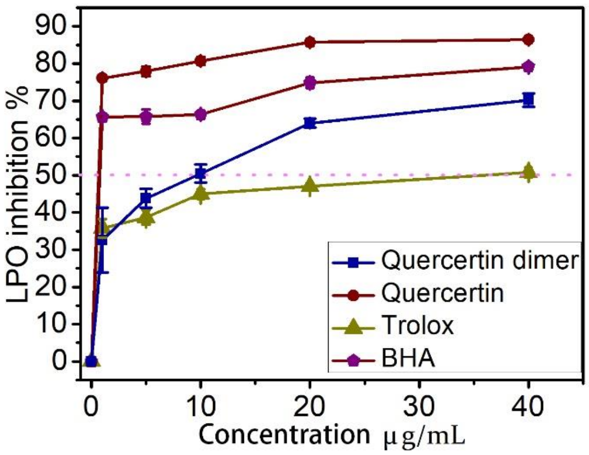

Figure s1.5. Concentration-response curves for quercetin, (±) quercetin Diels-Alder anti-dimer, Trolox and BHA in lipid peroxidation-scavenging measurement (linoleic acid emulsion method). Each value is expressed as the mean ± S, n = 3.

Table 5. The IC_{50} values of QDAD, quercetin, **Trolox** and **BHA** in lipid peroxidation-scavenging

	QDAD	Quercetin	Trolox	BHA
μg/mL	9.2±1.2	0.9±0.1	34.2±1.2	0.6±0.1
μM	15.5±1.2 [c]	2.2±0.02 [a]	136.0±12.3 [d]	3.0±0.1 [b,*]

The IC_{50} value is defined as the lowest concentration with 50% radical inhibition or relative reducing power, calculated by linear regression analysis, and expressed as the mean ± SD (*n* = 3). The linear regression was analyzed using Origin 6.0 professional software. The IC_{50} values in μM with different superscripts ([a] and [b]) in the same row are significantly different ($p < 0.05$). Trolox and ascorbic acid were used as the positive controls.

The data in Red are cited in Table 1 in the main text.
